# The prevalence of primary headache disorders and their associated factors among nursing staff in North China

**DOI:** 10.1186/1129-2377-16-4

**Published:** 2015-01-13

**Authors:** Yan Wang, Jingdan Xie, Fei Yang, Shiwen Wu, Hebo Wang, Xiaolan Zhang, Hua Liu, Xin Deng, Shengyuan Yu

**Affiliations:** International Headache Center, Department of Neurology, Chinese PLA General Hospital, Fuxing Road 28, Haidian District, Beijing, 100853 China; Department of Neurology, The General Hospital of Chinese Armed Police Forces, Beijing, 100039 China; Department of Neurology, Hebei General Hospital, Shijiazhuang, 050051 Hebei Province China

**Keywords:** Prevalence, Headache, Migraine, Tension-type headache, Chronic daily headache, Nursing staff

## Abstract

**Background:**

Epidemiological data on the prevalence of headache in nursing staff in Mainland China are lacking. We therefore performed a study to assess the prevalence of headache, and factors associated with headaches, in nursing staff in three hospitals in North China.

**Methods:**

Stratified random cluster sampling was used to select 1102 nurses from various departments in three hospitals. A structured questionnaire was used to collect epidemiological data, headache characteristics and associated factors.

**Results:**

The response rate was 93.0%. Among nursing staff, the 1-year prevalence of primary headache disorders was 45.3%, of migraine 14.8% (migraine with aura 3.4%, migraine without aura 11.4%), of tension-type headache (TTH) 26.2%, of chronic daily headache (CDH) 2.7%. Multivariate analysis showed that seniority (≥5 years) was a risk factor for migraine (OR 2.280), obesity (BMI ≥ 25) was a risk factor for TTH and CDH (OR 1.684 and 3.184), and age (≥40 years) was a risk factor for CDH (OR 8.455). Nurses working in internal medicine were more likely to suffer CDH than those in other departments. Working a greater number of night shifts was also associated with increased prevalence of headache.

**Conclusion:**

The prevalence of primary headache disorders in nurses is higher than that in the general population in China, and occupational factors may play an important role. Therefore, the prevalence of headache in nurses should be a focus of attention, and coping strategies should be provided. Such measures could contribute to improving patient care.

## Background

Primary headache, especially migraine and tension-type headache (TTH) are common in the general population worldwide [1, 2]. The current headache prevalence is 46% in the adult population worldwide [1]; Asians have a lower prevalence than European and North American populations due to racial differences [3, 4]. In a door-to-door population-based survey in China, the 1-year prevalence of primary headache disorders was 23.8%, and was higher in females [5]. Headache can affect work and other activities, with most migraine sufferers and around half of tension-type headache sufferers reporting limitation of activities during a headache attack [6, 7]. Due to the high prevalence of headaches and the associated disability, the presence of headaches in specific professional groups should be investigated.

Nursing staff, who are primarily female, experience a huge source of stress as a result of caring for suffering and dying patients, and through challenging physician-patient relationships, more easily to suffer headache than general population [8, 9]. Studies have explored nurses’ occupational stress and coping [10, 11] but no study has investigated the headache prevalence in nurses in mainland of China. Studies conducted in Taiwan and Japan revealed that the prevalence of headaches in nurses is higher than in the general population [8, 9]. There has been limited research in this area worldwide, and, at present, little is known about the prevalence of headache among nursing staff in Mainland China.

The purpose of this epidemiological study was to investigate the prevalence of primary headache and factors associated with headache among nursing staff in China. The diagnostic criteria were based on the International Classification of Headache Disorders, 3rd edition (beta version) (ICHD-3-beta) [12]. In addition, we evaluated the impact of nursing occupational factors on the prevalence of primary headache.

## Methods

### Ethics

The study protocol was approved by the Ethics Committee of the Chinese PLA General Hospital, Beijing. All participants provided written informed consent after receiving a detailed explanation of the purpose and design of the study.

### Questionnaire

Participants filled out a structured questionnaire to gather demographic and socioeconomic data, headache characteristics over the previous year, and occupation-related factors. The demographic and headache profile sections of the questionnaire were the same items as used in a Chinese national epidemiology study, and were validated for headache assessment and diagnosis in the general population [5, 13].

Demographic questions included age, ethnicity (Han versus Non-Han), marital status (Unmarried versus Married/Divorced, we merged the latter two as the number of divorced people was low), educational attainment (Junior college or lower versus University or above), body-mass index (BMI, graded as underweight, normal weight, overweight, obese); socioeconomic status including nursing specialty (internal medicine, surgical department and others), work seniority (<5 years versus ≥5 years), title (primary nurse, nurse practitioner, nurse-in-charge or above).

The headache profile section included items on headache duration, frequency, location, quality, intensity, aura, and characteristics of accompanying symptoms (nausea, vomiting, photophobia and phonophobia), and the impact of physical activity on headache. The diagnoses of migraine and TTH were made according to the criteria of ICDH-3-beta, respondents reporting headaches lasting more than 4 h per day on 15 or more days per month were given the label CDH and questioned on medication usage in order to identify medication-overuse headache (MOH) [14]. Trigeminal autonomic cephalalgia, other primary headaches and secondary headaches were not included in this questionnaire.

The working patterns of nursing staff are unique. We therefore assessed occupation-related factors, including work arrangements (rotational shifts) and number of night shifts (for those completing shift work).

### Sample and survey

The study was conducted in three 3A hospitals in North China from December 2013 to June 2014. The hospitals were the Chinese PLA General Hospital, The General Hospital of Chinese Armed Police Forces and the Hebei General Hospital. We adopted a stratified random cluster sampling method. In each hospital, we randomly selected eight clinical departments from which all nurses were invited to participate. Each participant was interviewed face-to-face by neurologists who were systematically trained with the ICHD-3-beta tool and the survey, then completed and retrieved the structured questionnaire. Participants who reported headache were followed up in a telephone interview to confirm diagnosis. Prior to this, a pilot study surveyed one department in each hospital to test the epidemiological methods.

### Statistics

Data were processed using EpiData 3.1 and analysed using SPSS 17.0. Continuous variables which did not comply with the normal distribution were summarised as median, and categorical variables as numbers and percentages. Chi-square tests were used to compare the distributions of categorical variables between groups. Multivariate logistic regression was applied to identify odd ratios (ORs) with 95% confidence intervals (CIs) for different types of headache, according to social-demographic characteristics. Statistical significance was set at p < 0.05.

## Results

Among the 1102 nurses invited to participate, 58 declined to complete of the survey, and 18 submitted incomplete questionnaires. The response rate was 93%. 1023 respondents completed the survey, all female. The age ranged from 20-57 years with a median of 27 years.

The 1-year prevalence of primary headache was 45.3% (95% CI 42.4-48.4%), with 14.8% (95% CI 9.2-20.4%) experiencing migraine (3.4% for migraine with aura, 11.4% for migraine without aura), 26.2% (95% CI 21.1-31.3%) TTH, and 2.7% (95% CI 0-8.7%) CDH. Only 10 respondents reported two types of primary headache, and 5 had unclassifiable headache. Only 2 nurses were diagnosed with both CDH and MOH. The prevalence of TTH peaked during middle age (30-39 years) (30-39: 33%; 20-29: 24%; ≥40: 29.3%), while migraine and CDH increased gradually with age (migraine: 20-29: 13.8%, 30-39: 17.2%, ≥40: 18.7%; CDH: 20-29: 1.7%, 30-39: 3.4%, ≥40: 10.7%).

**Figure 1:**
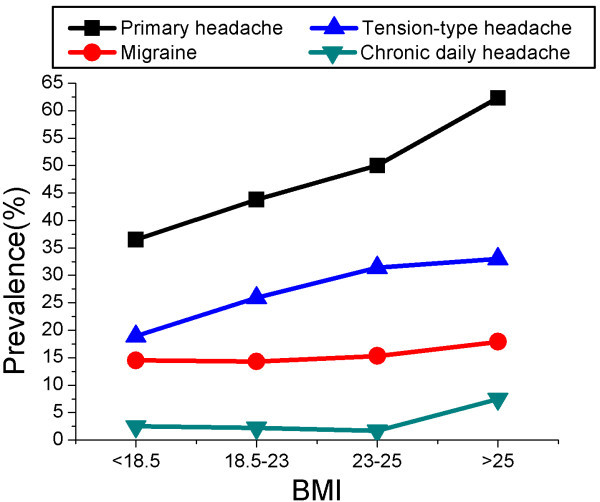
**The 1-year prevalence of different types of headache by BMI.**

The demographic data comparisons between different types of headaches and non-headache are shown in Table 1. Univariate analysis suggested that the prevalence of all three types of headache differed significantly with age, marital status, and seniority. Education was associated with the likelihood of experiencing headache but not with a particular kind of headache. Nursing specialty correlated with CDH, job title and BMI were linked to TTH and CDH but not migraine. Being married or divorced increased the probability of suffering all three headache types (migraine: married/divorced: 25.8%, unmarried: 18%, P < 0.05; TTH: married/divorced: unmarried: 38.6%, 27.4%, P < 0.01; CDH: married/divorced: 7.8%, unmarried: 2.6%, P < 0.01). More highly educated participants were prone to having more headaches, especially migraine (University or above: 25.3%, Junior college or lower: 18.9%, P < 0.05). Those with seniority of greater than 5 years were more likely to suffer all types of headache than were less-senior staff (migraine: seniority ≥5: 27.5%, seniority <5: 15.2%, P < 0.01; TTH: seniority ≥5: 37.1%, seniority <5: 27.8%, P < 0.01; seniority ≥5: 7.5%, seniority <5: 2.3%, P < 0.01). The prevalence of TTH and CDH was significantly higher in some roles (TTH: nurse 28.6%, nurse practitioner 33%, nurse-in-charge or above 42.8%, P < 0.05; CDH: nurse 2.9%, nurse practitioner 4.2%, nurse-in-charge or above 13.3%, P < 0.01). The prevalence of migraine and total headache did not significantly differ with work arrangements (day shift vs. rotating shift with day, evening and night shifts). However, nurses working day shifts were significantly more likely to suffer from TTH (TTH: day-shift 36.7%, rotating-shift 30%, P < 0.05).Figure 1 shows the trends in the prevalence of primary headache associated with BMI. The prevalence of TTH increased with increasing BMI. In migraine and CDH, the prevalence initially marginally decreased and then increased with increasing BMI. Participants classified as obese (BMI >25) had a significantly increased prevalence of all types of headache compared with those of a normal weight (migraine: OR = 1.86, 95% CI 1.04 to 3.34, P < 0.05; TTH: OR = 1.9, 95% CI 1.17 to 3.08, P < 0.01; CDH: OR = 5.14, 95% CI 2.21 to 11.99, P < 0.01).

**Table 1:** **The demographic characteristics comparisons between different types of headaches and non-headache among nursing staff**

Variable	Non-headache N	Total headache		Migraine		Tension-type headache		Chronic daily headache	
		N(%)	P value	N(%)	P value	N(%)	P value	N(%)	P value
**Total**	560	463(45.3)		152(21.3)		268(32.4)		28(4.8)	
**Age**			0.000		0.028		0.002		0.000
20-29	437	308(41.3)		103(19.1)		179(29.1)		13(2.9)	
30-39	92	111(54.7)		35(27.6)		67(42.1)		7(7.1)	
≥40	31	44(58.7)		14(31.1)		22(41.5)		8(20.5)	
**Nationality**			0.407		0.047		0.309		0.843(adjusted)
Han	535	440(45.1)		139(20.6)		260(32.7)		26(4.6)	
Non-Han	25	23(47.9)		13(34.2)		8(24.2)		2(7.4)	
**Marital status**			0.000		0.012		0.001		0.004
Unmarried	336	220(39.6)		74(18)		127(27.4)		9(2.6)	
Married/Divorced	224	243(52)		78(25.8)		141(38.6)		19(7.8)	
**Education**			0.009		0.044		0.073		0.067
Junior colleague or lower	356	257(41.9)		83(18.9)		153(30.1)		13(3.5)	
University or above	204	206(50.2)		69(25.3)		115(36.1)		15(6.8)	
**Nursing specialty**			0.825		0.559		0 .701		0.046
Internal Medicine	237	207(46.6)		63(21)		120(33.6)		18(7.1)	
Surgical Department	198	162(45)		60(23.3)		87(30.5)		8(3.9)	
Others	125	94(42.9)		29(18.8)		61(32.8)		2(1.6)	
**Seniority(year)**			0.000		0.000		0.004		0.003
<5	302	186(38.1)		54(15.2)		116(27.8)		7(2.3)	
≥5	258	277(51.8)		98(27.5)		152(37.1)		21(7.5)	
**Title**			0.002		0.559		0.017		0.001
Primary nurse	267	177(39.9)		54(16.8)		107(28.6)		8(2.9)	
Nurse practitioner	229	204(47.1)		75(24.7)		113(33)		10(4.2)	
Nurse-in-charge or above	64	82(56.2)		23(26.4)		48(42.9)		10(13.5)	
**Work arrangement**			0.145		0.82		0.046		0.067
Day-shift	186	174(48.3)		49(20.9)		108(36.7)		14(7)	
Rotating-shift	374	289(43.6)		103(21.6)		160(30)		14(3.6)

Then the above factors were analyzed by enter method of multivariate logistic regression (Table 2), revealing that seniority ≥5 years and BMI ≥25 remained independent risk factors for total headache, as did seniority ≥5 years for migraine, BMI ≥25 for TTH, age ≥40 years and BMI ≥25 for CDH. Nurses of other specialties were less likely to suffer CDH than internal medicine and surgical department nurses, but the number of other them was very low. Age for migraine and TTH, seniority for TTH and CDH, marital status and title for all three types of headache, work arrangement for TTH, and obesity for migraine were not identified as risk factors by multivariate logistic regression analysis.

**Table 2:** **Multivariable adjusted odds ratios (95% confidence interval) for total headache, migraine, tension-type headache (TTH), and chronic daily headache (CDH)**

	Total headache	Migraine	Tension-type headache	Chronic daily headache
**Age**				
20-29	Reference	Reference	Reference	Reference
30-39	1.166(0.751-1.809)	1.070(0.585-1.959)	1.242(0.742-2.078)	1.464(0.371-5.780)
≥40	1.401(0.667-2.944)	1.732(0.617-4.857)	1.043(0.438-2.482)	8.455(1.138-62.848)*
**Seniority (years)**(≥5 VS <5)	1.468(1.013-2.126)*	2.280(1.323-3.929)**	1.122(0.718-1.752)	3.724(0.890-15.579)
**Nursing specialty**				
Internal Medicine	Reference	Reference	Reference	Reference
Surgical Department	0.989(0.741-1.320)	1.121(0.740-1.699)	0.914(0.649-1.289)	0.464(0.187-1.152)
Others	0.848(0.605-1.188)	0.766(0.459-1.279)	0.998(0.676--1.474)	0.136(0.027-0.673)*
**BMI**				
Normal weight (18.5 to <23)	Reference	Reference	Reference	Reference
Underweight (<18.5)	0.804(0.557-1.160)	0.982(0.582-1.659)	0.704(0.447-1.110)	1.685(0.507-5.603)
Overweight (23 to <25)	1.082(0.719-1.626)	0.959(0.525-1.752)	1.177(0.736-1.883)	0.509(0.105-2.473)
Obese (≥25)	1.814(1.165-2.823)**	1.515(0.802-2.863)	1.684(1.011-2.806)*	3.184(1.116-9.089)*

*P < 0.05, **P < 0.01.

We investigated whether frequency of night shift affects the prevalence of headache by grouping nurses above or below the median of eight (see Table 3). Nurses working greater than eight night shifts were significantly more likely to suffer all types of headache than those working less than eight night shifts (Migraine: 29.4% *vs.* 18.9%; TTH: 35.5% *vs.* 28.1%).

**Table 3:** **The impact of number of night shifts per month on the prevalence of different types of primary headache**

	Total headache		Migraine		Tension-type headache	
	Prevalence (%)	OR (95% CI)	Prevalence (%)	OR (95% CI)	Prevalence (%)	OR (95% CI)
**Night shift number**		**1.59(1.13-2.23)**		**1.79(1.12-2.85)**		**1.41(0.93-2.12)**
≤8	**40.5**		**18.9**		**28.1**	
>8	**51.9**		**29.4**		**35.5**	

## Discussion

This study is the first to assess the prevalence of headaches among nursing staff in Mainland China. We found that the 1-year prevalence of primary headaches was 45.3% (95% CI 42.4-48.4%). This is higher than the prevalence in females in the general population in Mainland China (36.8% [95% CI 34.9-38.7%]) according to data from a previous nationwide population-based study conducted using the same screening questionnaire [5]. Our findings were similar to those of previous studies in Taiwan and Japan, which also found that the prevalence of primary headache was higher in nursing staff compared to the general population. In Taiwan, the prevalence of primary headache was 49.6% [9, 15], while in Japan, the prevalence of recurrent headache was 40.9% [8]. Compared to the general population, nurses, as a special occupational group with basic medical knowledge, took medicine more rationally, which could be the reason for low prevalence of MOH. This side-fact indicated the importance of popularization of medical knowledge. Multivariate analysis revealed that seniority of greater than 5 years was a risk factor for migraine; obesity was a risk factor for TTH; and age, obesity, and internal medicine specialty were risk factors for CDH.

The 1-year prevalences of migraine, TTH and CDH in our study were 14.8%, 26.2% and 2.7%. These were higher than reported in the general female population in which the prevalence was 12.8% for migraine, 14% for TTH, and 1.4% for CDH. The difference in prevalence between the two populations was greatest for TTH. The reason for this might be that nurses were exposed to a greater number of occupational stressors due to a large workload, poor work environment, and difficult patients [8, 10, 11, 16, 17]. High work stress is a risk factor for primary headache, especially TTH [8, 18–23]. The previous national population-based study in Mainland China revealed that the prevalence of TTH and migraine peaked during middle age (40-49 years), and CDH gradually increased with age [5]. Japanese and Taiwan studies have reported that the highest prevalence of migraine in females occurs at the age of 30-39 years [14, 24]. The peak in prevalence of migraine in our study was 40-49 years; this is consistent with the population-based study, but later than in the two other Asian studies. Takeshima et al. reported that the peak age for TTH in Japan was 40-59 years [25]. Studies conducted in Malaysia and Hong Kong reported younger peak ages of 16-35 and 25-34 years, respectively [26, 27], which were similar to our findings (30-39 years). The younger peak age in TTH compared to the Japanese study might be because of the relatively large proportion of young nurses. The prevalence of CDH increased as individuals aged, which is consistent with the findings of the general population study in Mainland China.

In the present study, a trend indicated that higher prevalence of TTH was associated with higher BMI; this is contrary to the commonly held belief that BMI is a risk factor for the frequency, but not prevalence, of headache [28, 29]. Although previous studies suggested that migraine prevalence was significantly associated with obesity in reproductive-age individuals [30, 31], the relationship between obesity and prevalence of episodic tension-type headache was first revealed in our clinic-based study, which found that obese participants were almost twofold more likely to have TTH than the healthy weight controls. As data regarding the association between TTH and obesity are limited, the underlying reasons are unclear. The difference may be the result of different BMI classifications; we adopted the guideline specifically for the Asia-pacific population [32], which define obesity as BMI ≥25 kg/m^2^, and did not separately calculate morbid obesity in nursing staff as the numbers were low. We believe that the characteristics of our study group may be in part responsible for this difference. Nurses, who are predominantly female, tend to pay more attention to their own appearance and might develop anxiety and depression if obese, this anxiety and depression may lead to TTH [23, 33]. In addition, obesity and TTH have an overlapping pathophysiology. Low serotonin levels may increase food intake and development of obesity, and are also thought to play an important role in TTH [34]. Levels of several inflammatory mediators, including IL-1, IL-6, and tumour necrosis factor (TNF)-α, are increased in obese individuals [35], and these have been proposed to contribute to the development of TTH [36, 37]. Multivariate logistic regression indicated no significant association between migraine and obesity, which was not consistent with previous studies [29, 38]. The difference in findings might be due to the small sample size in our study. Obese participants (BMI > 25) were more than threefold more likely to suffer CDH than the participants of a healthy weight. This conclusion is consistent with previous reports of an association between obesity and the frequency of episodic headache [29]. Individuals with episodic headache and obesity develop chronic daily headache (CDH) at more than fivefold the rate of normal-weight individuals [39].

It revealed that the nurses of other specialties were less likely to suffer CDH than internal medicine and surgical department nurses, however, there were only 2 nurses of other specialities suffering CDH, so this finding maybe not so believable. In our study, seniority of greater than 5 years was significantly associated with a greater prevalence of primary headache, especially migraine, indicating that occupational factors affect the prevalence of headache among nursing staff. As the seniority increased, nurses would shoulder a greater work pressure and face more complicated personal relationship, which all could lead to the attack of migraine. Among the rotating-shift group, working greater than eight night shifts per month was associated with a higher incidence of primary headache. In previous observational studies, it was found that night shift work was associated with an increased risk of cancer and cardiovascular disease [40–42], but the effect of night shift work on the risk of headache had not been assessed previously. More night shift work may lead to increased sleep disturbance and chronic fatigue, which are triggers of headaches.

Our study had several strengths. First, it was the first study in Mainland China to assess the prevalence and associated factors of primary headache in nursing staff. Second, the random cluster sampling method utilised combined with the high response rate eliminated selection bias. Furthermore, the diagnosis of headache met the latest ICDH-3-beta guidelines, and a follow-up telephone interview conducted by a neurologist guaranteed the accuracy of the diagnosis.

This study had several limitations. Despite our ability to control for a large number of potential confounders, we could not assess these comprehensively, and did not investigate other psychosomatic diseases that might be confounding variables. We did not investigate lifestyle factors, such as lack of exercise, smoking, drinking, sleeping late, all of which could increase the prevalence of headache. We used a structured questionnaire to collect data, we can’t know in detail the headache profile before, then it is difficult for us to judge what types of headache the chronic daily headache was transformed from, so we cannot distinguish the chronic migraine from chronic tension-type headache. The sample was relatively small, involving only three hospitals and 1023 nurses in North China, therefore the number of outcome events in some subgroups, especially chronic daily headache with low prevalence, is small. Further work is needed.

## Conclusion

This epidemiological study was performed to assess the prevalence of primary headache in nursing staff in Mainland China. The prevalence was high, which suggests that occupational health problems of nurses should be focused upon. Awareness and avoidance of trigger factors can not only decrease the frequency of headache but also reduce the possibility of chronic headache and medication overuse, guaranteeing the working health of nurses and thus improve their output. Greater attention to, and better management of, primary headache among nursing staff could improve health care in China.

## Abbreviations

TTH: Tension type headache
CDH: Chronic daily headache
MOH: Medication-overuse headache
ICHD-3-beta: International Classification of Headache Disorders, 3rd edition (beta version).
